# Analysis of nonsynonymous SNPs in candidate genes that influence bovine temperament and evaluation of their effect in Brahman cattle

**DOI:** 10.1007/s11033-024-09264-4

**Published:** 2024-02-07

**Authors:** Gilberto Ruiz-De-La-Cruz, Ana María Sifuentes-Rincón, Francisco Alejandro Paredes-Sánchez, Gaspar Manuel Parra-Bracamonte, Eduardo Casas, David G. Riley, George A. Perry, Thomas H. Welsh Jr., Ronald D. Randel

**Affiliations:** 1 Laboratorio de Biotecnología Animal, Centro de Biotecnología Genómica, Instituto Politécnico Nacional, Reynosa, Tamaulipas, 88710 México; 2 Unidad Académica Multidisciplinaria Mante, Universidad Autónoma de Tamaulipas, El Mante, Tamaulipas, 89840 México; 3 National Animal Disease Center, Agricultural Research Service, United States Department of Agriculture, Ames, IA 50010 USA; 4 Department of Animal Science, Texas A&M University, College Station, TX 77843 USA; 5 Texas A&M AgriLife Research, Overton, TX 75684 USA

**Keywords:** Behavior, Candidate genes, Modelling, Amino acid change, Serotonin receptor

## Abstract

**Background:**

Temperament is an important production trait in cattle and multiple strategies had been developed to generate molecular markers to assist animal selection. As nonsynonymous single nucleotide polymorphisms are markers with the potential to affect gene functions, they could be useful to predict phenotypic effects. Genetic selection of less stress-responsive, temperamental animals is desirable from an economic and welfare point of view.

**Methods and results:**

Two nonsynonymous single nucleotide polymorphisms identified in HTR1B and SLC18A2 candidate genes for temperament were analyzed *in silico* to determine their effects on protein structure. Those nsSNPs allowing changes in proteins were selected for a temperament association analysis in a Brahman population. Transversion effects on protein structure were evaluated *in silico* for each amino acid change model, revealing structural changes in the proteins of the HTR1B and SLC18A2 genes. The selected nsSNPs were genotyped in a Brahman population (*n* = 138), and their genotypic effects on three temperament traits were analyzed: exit velocity, pen score, and temperament score. Only the SNP rs209984404-HTR1B (C/A) showed a significant association (*P* = 0.0144) with pen score. The heterozygous genotype showed a pen score value 1.17 points lower than that of the homozygous CC genotype.

**Conclusion:**

The results showed that *in silico* analysis could direct the selection of nsSNPs with the potential to change the protein. Non-synonymous single nucleotide polymorphisms causing structural changes and reduced protein stability were identified. Only rs209984404-HTR1B shows that the allele affecting protein stability was associated with the genotype linked to docility in cattle.

**Supplementary Information:**

The online version contains supplementary material available at 10.1007/s11033-024-09264-4.

## Introduction

The identification of genetic variations associated with economically relevant traits is one of the goals for genomics applied to production systems. Single nucleotide polymorphism (SNP)-based analyses have proven to be technically and biologically efficient for systematically exploring the genetic architecture of complex traits such as bovine temperament [1, 2]. Temperament is a complex animal behavior trait defined by the interaction of past experiences and environmental and genetic factors [3] and is considered an economically relevant trait associated with productive traits such as growth, health, and quality carcasses [4, 5]. Cattle temperament tests include objective and subjective methods [6]. Numerous strategies, such as candidate gene resequencing, quantitative trait loci, and whole genome analysis, have been used to decipher the genetic structure of cattle temperament. Currently, numerous genes are orthologous between hu-mans and cattle [7], and the variants that are associated with the temperament of cattle are in genes region involved in disorders and diseases of human behavior [8].

The candidate genes associated with temperament in cattle include neurotransmitter receptors, such as a family of dopamine and serotonin receptors [9]. The dopamine and serotonin receptors are associated with characteristics of anxiety, depression, aggression, and stress response in humans [10]. For example, the serotonin receptor gene HTR1B has been related to human mental disorders and associated with aggressive behavior in dogs [2]. Genes such as SORCS3 and SESTD1 have been associated with cattle temperament; they have activities such as the function modulator postsynaptic of synaptic depression, fear extinction, and signal transmission capability [11]. Various genomic regions of dairy and beef cattle have associated candidate genes with temperament, such as the C8B, POMC, MIPOL1, and SLC18A2 genes [12].

Nonsynonymous SNPs (nsSNPs) are of particular interest since they have the potential to affect the structure and functionality of the protein encoded by the gene in which they are located [13]. The SLC11A2 gene in Zebu cattle has an nsSNP that impacts protein structure and has functional consequences [14]. Moreover, in the Charolais breed, it has been found that the SLC18A2 gene harbors an nsSNP with a temperament effect, but it has yet to been analyzed whether the nsSNP causes a modification in the protein structure [9]. We aimed to identify nsSNPs located in candidate genes for temperament in cattle and, by an *in silico* analysis, prioritize the nsSNPs affecting the protein structure to analyze their phenotypic effect on temperament traits measured by exit velocity (EV), pen score (PS), and temperament score (TS) in a population of Brahman cattle.

## Materials and methods

### *In silico* analysis and gene selection

Considering the interaction network of 26 genes related to temperament traits reported by Garza-Brenner et al. [9], 17 genes harboring nonsynonymous SNPs were selected and included in an in silico analysis (Table 1).

**Table 1:** List of candidate genes related to temperament and the number of SNPs per gene

Gene	ID	nsSNPs
*ADRA2A* Adrenergic adrenoceptor alpha-2 A-receptor	282135	7
*ADRA2B* Adrenergic adrenoceptor alpha-2B-receptor	516422	13
*DRD1* Dopamine receptor D1	281125	2
*DRD2* Dopamine receptor D2	281126	3
*DRD3* Dopamine receptor D3	537043	2
*DRD5* Dopamine receptor D5	526221	1
*FOSFBJ* Murine osteosarcoma viral oncogene homologous	280795	1
*HTR1B* 5-hydroxytryptamine receptor 1B	317707	2
*HTR2A* 5-hydroxytryptamine receptor 2 A	407230	4
*HTT* Huntingtin	615059	14
*MAOA* Monoamine oxidase A	281293	7
*MAOB* Monoamine oxidase B	338445	1
*PNMT* Phenylethanolamine N-methyltransferase	281413	4
*POMC* Proopiomelanocortin	281416	4
*SLC18A2* Solute carrier family 18-member 2	282471	1
*TDO2* Tryptophan 2,3-dioxygenase	530397	1
*TH* Tyrosine hydroxylase	280707	3

Gene: column with abbreviation and name genes; ID: identifier number according to National Center for Biotechnology Information; nsSNPs: non-synonymous single nucleotide polymorphisms number

The candidate genes in the study belong to different gene families, including adrenergic, serotonin, and dopamine receptors, which are responsible for regulating the activity of these neurotransmitters in the brain. Additionally, there are groups of enzymes, such as monoamine oxidase, tyrosine, tryptophan, and phenylethanolamine, which are involved in synthesizing and metabolizing these neurotransmitters. Other candidate genes are hormone precursors, vesicular transporters, and homologous genes associated with human diseases. Homologous genes are genes similar in structure and function to other genes and are often associated with similar diseases or conditions.

### Protein templates

The amino acid sequence of the candidate genes were searched in the National Center for Biotechnology Information (NCBI) public database (https://www.ncbi.nlm.nih.gov/ (accessed on 15 March 2020)), using the sequences of Bos taurus genome version ARS-UCD 1.3. Upon analyzing sequence genes, it was found that the NCBI database lists isoforms for dopamine receptor D1 (DRD1), dopamine receptor D2 (DRD2), dopamine receptor D3 (DRD3), huntingtin (HTT), proopiomelanocortin (POMC), and tyrosine hydroxylase (TH) genes. To ensure the general accuracy of our analysis, we specifically selected sequences with access and notation of NP_. In cases where a gene lacked a notation, we utilized the XP isoform presented on the Genome Data Viewer (Table 2). It is worth noting that the DRD1 isoform sequences were identical, while DRD2, DRD3, POMC, and TH genes had a notation of NP_. As for the HTT gene, we referenced XP_024849486.1 due to its listing on the Genome Data Viewer. Lastly, was edited one copy of the sequence to include the nsSNP.

**Table 2:** Genes reported with protein isoforms

Gene	Accession	Annotation
DRD1	281125	NP_776467.1
DRD2	281126	NP_776468.1
DRD3	537043	NP_001179824.1
HTT	615059	XP_024849486.1
POMC	281416	NP_776576.1
TH	280707	NP_776309.1

### Sequence 3D modeling

In order to perform an analysis of the original and modified sequences, they were first uploaded in FASTA format to the SWISS-MODEL online tool [15] (https://swissmodel.expasy.org/). This tool utilizes a multi-step process to generate accurate models of protein structures. First, it identifies structural templates that are similar to the target sequence. Then, it aligns the target sequence with the template structure to ensure a proper fit. Next, it builds the model based on the alignment and other relevant data. Finally, it evaluates the quality of the model to ensure that it is accurate and reliable. This comprehensive approach ensures that the resulting models are of the highest quality and can be used confidently in further analysis and research.

The models’ selection began with those with a high global model quality estimation (GMQE) value, followed by those within the 0.4-1 range suggested by SWISS-MODEL. The models generated had a .pdb extension and were evaluated on the RAMPAGE server [16] using Ramachandran plots. These plots allowed the analysis of the geometric distribution of the models and each residue, evaluating the torsion of the angles (phi φ and psi ψ) from different combinations and the possible conformations according to steric obstacles. A model was considered successful if it had a distribution of amino acids in favorable regions of percentages starting at 89% and > 90% for a good model. This thorough evaluation process ensured that only the most accurate and reliable models were selected for further analysis.

### Visualization and selection of models

The models were visualized and compared using PyMOL™ v.2.2.0 software from Schrodinger, LLC [17]. To do this, the outputs of the modeling process in the .pdb extension were uploaded in PyMOL. The models were then subjected to a superimposition, and sequence alignment was performed using the -align operation between the original and modified models. The resulting structures were displayed in a combination of colors, with perfectly superimposed structures appearing in the same color and any structural differences being displayed in independent colors.

When found models with secondary structures with independently colors that shows structural differences were selected for next analysis, and the models with perfectly superimposed structures were excluded.

### Prediction of protein stability change

To determine whether a protein is stable or not, we used I-Mutant 2.0 [18] to calculate the change in Gibbs free energy. I-Mutant 2.0 is an automated web server that uses a support vector machine to estimate the effects of amino acid substitutions and calculate the free energy change value of the residue position. This value is represented by DDG, which stands for delta delta Gibbs and indicates the unfolding Gibbs free energy value. If the DDG value is greater than 0 (DDG > 0), it means that the protein is more stable. On the other hand, if the DDG value is less than 0 (DDG < 0), it means that the protein is less stable.

### Genotyping and association analysis of nsSNPs

#### Biological samples

The study conducted in this research followed the Guide for the Care and Use of Agricultural Animals in Research and Teaching 2010, which ensures the ethical treatment of animals used in research and teaching. The study was approved by the Texas A&M University Animal Care and Use Committee AUP 2002 − 315, which is responsible for reviewing and approving animal research protocols.

The data collection methods and population were previously described in [19]. The Brahman samples used in the study were ear tissues collected from 64 males and 74 females born between 2002 and 2017 at the Texas A&M AgriLife Research and Extension Center in Overton, Texas.

The evaluations were recorded for calves Brahman at weaning, all the animals had their temperament assessed by three traits: exit velocity (EV), pen score (PS), and temperament score (TS). The EV is the velocity at which an animal travels 1.8 m after being released from a cattle chute and is calculated with infrared sensor data (FarmTek Inc., North Wylie, TX, USA). Animals with higher speeds represent an unfavorable temperament, while animals with lower speeds represent animals with a docile temperament [6, 20]. The PS is a subjective test that evaluates behavior when animals are confined inside a pen. An individual PS was assigned to each animal by a single trained evaluator when a score of 1 indicating calm and 5 indicating aggressive behavior [21].

Finally, TS is an average of the PS and EV [TS = (PS + EV)/2]. PS and EV were combined to create a new measure of temperament that compensates for the weaknesses of its wholly objective and subjective components. PS evaluates aggressive behaviors, but its subjective quality makes comparisons difficult. EV provides a more consistent general evaluation of temperament across different environments. PS and EV can be averaged to calculate a TS and represented with standard deviation value (SD), providing a more useful measure of temperament to the cattle [22].

#### Genotyping

The DNA extraction process was performed from 25 mg of ear tissue samples using the GenElute Mammalian Genomic DNA extraction kit (Cat G1N350, Sigma-Aldrich Co. LLC, St Louis, Missouri, USA) protocol. The extracted DNA was then genotyped using the Sequenom MassARRAY® platform (GeneSeek, Inc., Lincoln, NE, USA). Two SNPs were genotyped using this platform. The first SNP was rs209984404 of HTR1B, which is located at genome position 17091682. The rs209984404-SNP has an allelic change of C > A, resulting in an amino acid change of alanine to serine on residue 83. HTR1B is a gene that encodes a G protein-coupled receptor involved in regulating serotonin levels in the brain. The second SNP was rs110365063 of SLC18A2, located at genome position 37565104, with an allelic change of G > A, that provide an amino acid change of alanine to threonine on residue 187. SLC18A2 is a gene that encodes a vesicular monoamine transporter that transports monoamine neurotransmitters, such as dopamine and serotonin, into synaptic vesicles.

#### Statistical analysis

To investigate the relationship between genotype and temperament traits, an association analysis was conducted using the general linear procedure (GLM) from SAS® software ver. 9.4. (SAS Statistical Analysis System, Cary, NC, USA). A Shapiro-Wilk test was performed to verify normality. The GLM is a statistical method that allows for the analysis of continuous response variables in relation to one or more predictor variables. In this case, the predictor variable was genotype, while the response variable were the temperament traits (EV, PS, and TS). The model used was as follows:

$${Y_{ijk}}=\mu +{A_i}+{S_j}+{G_k}+{\varepsilon _{ijk}}$$

The statistical model analyzes three variables: EV, PS, and TS. The general mean was represented by the symbol µ, while the fixed effects were represented by *A*_*i*_, *S*_*j*_, and *G*_*k*_. *A*_*i*_ represented the fixed effect of year of birth, *S*_*j*_ represented the fixed effect of sex, and *G*_*k*_ represented the fixed effect of nsSNP genotype as a qualitative variable. The random residual error was represented by *ε*_*ijk*_. To identify significant effects with a confidence level of *P* < 0.05, the least-squares mean of the genotypes was compared using the predicted differences (PDIFF) statement, which applied a Tukey‒Kramer adjustment. The present study was designed to ascertain nominal significant differences with the minimal number of experimental units.

## Results

### Protein modeling

After the *in silico* analysis of nsSNPs, those producing protein structure changes based on the quality and inclusion of amino acid residues in the model as well as the effect of protein stability were located at the candidate genes HTR1B and SLC18A2 (Table Supplementary 1). These nsSNPs showed changes between models caused by the alternative alleles of the nsSNPs analyzed. The general characteristics of changes for the modeling analysis are described in Table 3. All the models with changes had an acceptable-to-high modeling quality according to the GMQE parameters.

**Table 3:** Characteristics of the proteins and reliability parameters of modeling

Gene	SNP ID	Modeling Quality		Structural changes	nsSNP position amino acid
		Original	Modified		
*HTR1B*	rs209984404	GMQE 0.75	GMQE 0.75	Changes in the alpha-helix structure	A83S
*SLC18A2*	rs110365063	GMQE 0.44	GMQE 0.44	Changes in the alpha-helix structure	A63T

Gene: abbreviation of gene name; SNP ID: Ensembl database identifier for SNP; Modeling Quality: column with quality values for both models; GQME: model quality value for SWISS-MODEL; Structural changes: position in secondary structure of protein; nsSNP position amino acid: amino acid residue of change, first letter corresponds of reference amino acid, number of residue position, and last letter the amino acid alter-native

**Fig. 1:**
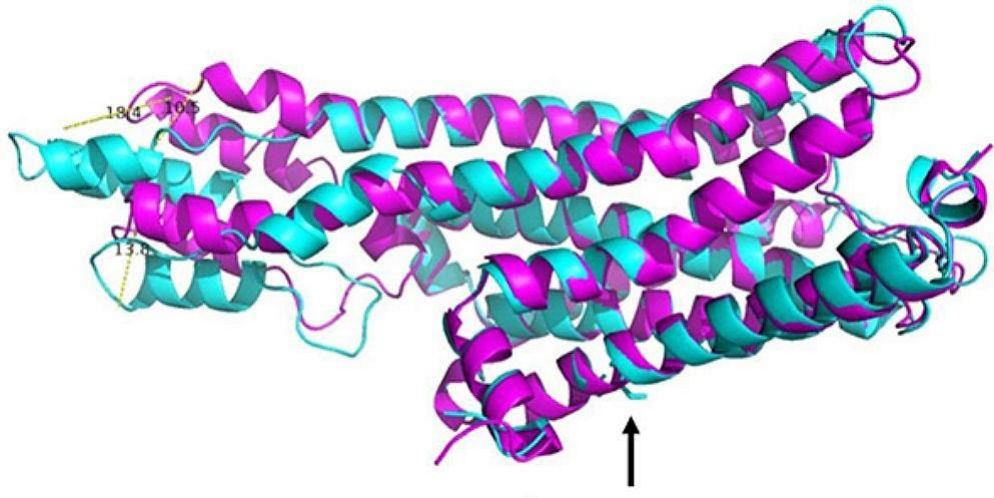
Superposition and alignment of models for HTR1B-transversion g.17091682:C > A. The superimposed segments show a combination of magenta and cyan, while the regions with structural differences show a single colour. The left side shows three distances among amino acid residues that show a structural change involving a group of loop structures and α-helices, while the arrow shows the residue change position

The serotonin receptor model of HTR1B with the transversion (g.17091682:C > A) at residue 83 results in the amino acid change of alanine to serine. Even though both amino acids are low molar mass (i.e., small), they differ in their chemical properties: alanine is hydrophobic whereas serine is a polar amino acid. Structural differences were evident during the superposition and alignment procedure (Fig. 1). The models showed different distances in three alpha-helices with values of 18.4, 10.5, and 13.8 Å, respectively.

**Fig. 2:**
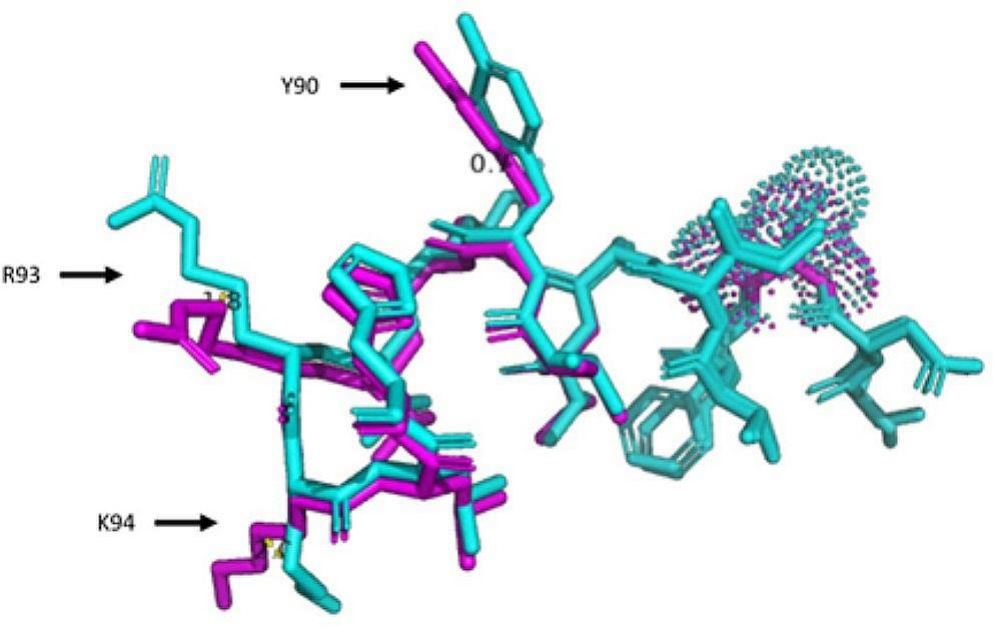
Visualization of residues 83 to 96 in stick form of the HTR1B gene. The cloud of dots at amino acid 83 indicate the site of the change, and arrows point to amino acids 90, 93 and 94 with structural changes

The change of alanine to serine at position 83 in HTR1B also promoted structural changes in the nearby residues, i.e., residue 90 of tyrosine showed a 0.7 Å change in the spatial orientation of its amino acid ring, residue 93 of arginine displayed a 1.8 Å relocation of the orientation of the amino group, and residue 94 of lysine showed orientation changes in its side chain (Fig. 2).

**Fig. 3:**
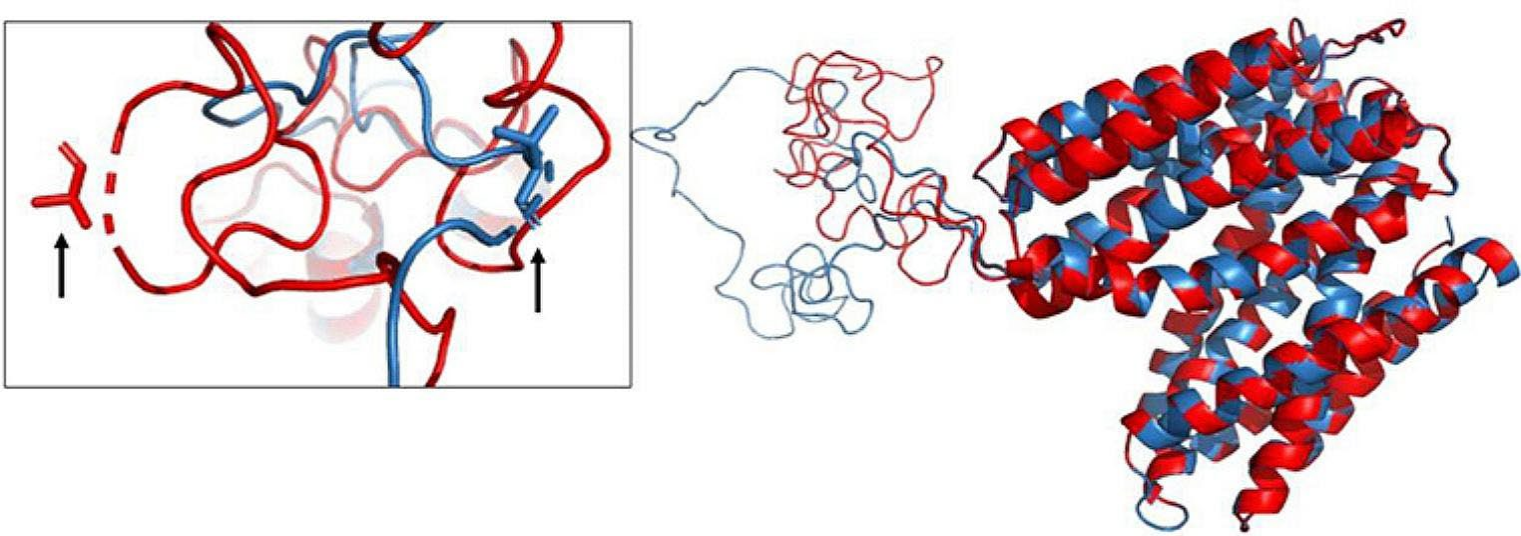
Superposition and alignment of SLC18A2 models. The superimposed segments combine red and blue colors, while the regions with structural differences show a single color. The left square shows a magnified view of the amino acid residue with a structural change in the loop structure; the arrows show the residue change position

The model of SLC18A2 with a nsSNP (g.37565104:G > A) changes residue 63 from ala-nine to threonine in a loop region (Fig. 3). The global structure of the protein showed differences during the superposition of models and a change in the number of alpha-helices (twelve in the original model and thirteen in the modified model) because alanine is a tiny, hydrophilic amino acid with a reactive chain, whereas threonine is a hydroxylated amino acid.

### Prediction of protein stability changes upon mutations

The alternative alleles in both analysed genes showed a decreased free energy change value (DDG) due to amino acid substitution; the change A > S in the HTR1B model had a -0.58 Kcal/mol, while the A > T substitution in the SLC18A2 model had a -1.24 Kcal/mol. These values are considered predictors of decreased protein stability.

### Effect of nsSNPs on temperament measurements in the Brahman population

The SNPs rs209984404-HTR1B and rs110365063-SLC18A2 identified by their effects on the protein structures were evaluated to determine their association with temperament values: EV, PS, and TS. The nsSNP rs209984404-HTR1B had a mean of 3.02 ± 0.26 m/s for EV, 3.03 ± 0.22 points to PS, and 3.03 ± 0.21 SD to TS, for the CC genotype. While CA genotype the means were 3.01 ± 0.56 m/s, 1.86 ± 0.48 pints, and 2.43 ± 0.44 SD, respectively. With a significant effect (*P* < 0.0144) on PS (Table 4). The genotyped population had only two genotypes for rs209984404-HTR1B, and animals with the heterozygous CA genotype had PS values 1.17 points lower than those of the homozygous CC genotype (Table 4).

**Table 4:** Association analysis and genotype distributions in the Brahman population

Gene	SNP ID	n	Genotype	LSM ± SE		
				EV	PS	TS
*SLC18A2*	rs110365063			(0.666)	(0.289)	(0.749)
		2	AA	6.36 ± 1.48	2.21 ± 1.31	4.28 ± 1.18
		18	AG	2.94 ± 0.54	3.17 ± 0.48	3.06 ± 0.43
		117	GG	3.01 ± 0.26	2.9 ± 0.23	2.96 ± 0.21
*HTR1B*	rs209984404			(0.97)	(0.014)	(0.17)
		117	CC	3.02 ± 0.26	3.03 ± 0.22 ^a^	3.03 ± 0.21
		19	CA	3.01 ± 0.56	1.86 ± 0.48 ^b^	2.43 ± 0.44

Gene: abbreviation of gene name; SNP ID: Ensembl database identifier for SNP; n: number of samples; Genotype: column with genotypes found in the population; LSM ± SE = lasts squares means and standard error; EV = exit velocity; PS = pen score; TS = temperament score. ^a,b^ Values with different superscripts differ significantly at *P* < 0.05

## Discussion

### Analysis of the impact of nsSNPs in protein models

From the evaluated nsSNPs panel, two of them had changes in protein structure. The change of alanine to serine residue 83 at rs209984404-HTR1B located in an α-helix had a structural effect on the next amino acid residues for the strong bonds and highest amino acid interactions of alpha-helix [23]. The structural modification in the α-helix due to the change in the properties of the amino acid caused an energy difference that can be seen in the global alignment of the models, coinciding with what was reported by Qureshi et al. [24], where an amino acid change from a nsSNP causes energy fluctuations up to 300 residues in the α-helix, altering the topology of the protein.

The change in rs110365063-SLC18A2 is in the loop region, producing spins between secondary structures [23]. The loop structures share some amino acid bounds, and the change in the physicochemical properties of nsSNPs has provided a stability change shown in the differences in the global structures. Abduljaleel et al. [25] report that changes in the loop structure of a protein affect the energy stability of residues and have an importance for interaction with other proteins. The changes in the protein structure by a nsSNP lead to changes in properties and binding energy between amino acids so that in both genes, changes in intramolecular interactions can be expected [26]. The intramolecular interactions can explain a variation in protein function, in addition to having a hydrophilic change that may result in differences in interaction with the aqueous cellular environment [27].

The substitutions in the loop region of SLC18A2 have the greater decrease in stability compared to those in the alpha-helix of HTR1B since, in the loop region, the amino acids have fewer interactions. However, for both genes, the changes in amino acid residues would modify protein function by altering free energy values [28]. According to our *in silico* analysis, HTR1B could maintain the passage of neurotransmitters but modify receptor performance [29, 30], and SLC18A2, as a transporter, could lose its interaction with other proteins, implying a difference in performance and altering the flow of neurotransmitters in cells [2]. Yue et al. [31] indicated that in 80% of cases, nsSNPs tend to reduce stability and therefore incorrectly fold, leading to inefficient stability of the protein.

### Association of nsSNPs with temperament

The effect of rs110365063-SLC18A2 and rs209984404-HTR1B was further tested by association analysis with temperament traits in Brahman cattle. Analysis showed that the rs209984404-HTR1B gene is significantly associated (*P* = 0.0144) with PS, which measures the animal response from a small group within a pen to human proximity, reflecting its fear or aggressiveness [32]. The evaluated population presented only two genotypes, and it can be inferred that the animals with the CC genotype had greater values of PS than those with the CA genotype. The HTR1B receptor gene has been previously studied by Garza-Brenner et al. [9] in an association analysis of temperament traits evaluated in Charolais cattle, and the authors did not find an association of the SNPs located at the HTR1B receptor gene with temperament traits. The HTR1B receptor has been linked to mental disorders in humans, and members of the HTR receptor family (1 A, 1B, 2 A) harbor polymorphisms that have been associated with aggressive behavior in dogs [2, 10]; in addition, in humans, HTR1B inactivation reduces anxiety levels, a behavior disorder resulting from a stress event [33].

According to Dutta et al. [34], the HTR1B gene is widely conserved across mammalian species; thus, based on previous insights into how HTR1B functions in stress pathways, it could also be assumed to be functional in cattle. Reported alterations in the function of HTR1B lead to reduced stress levels [33, 35]. Here, we found that the allelic change of nsSNP rs209984404-HTR1B has an effect on PS, and those animals carrying the allele that modifies the protein favored docile temperament-PS values. Nevertheless, although the association was identified, the sample size and the absence of a homozygote genotype do not allow the nsSNP to be considered yet as a selection marker. These results allow us to propose studies focused on the search for linkage disequilibrium with other variants forming haplotypes that allow them to be included in selection strategies. An increase in the number of observations and functional evidence are needed to confirm the proposed role of rs209984404-HTR1B in cattle temperament. Through the strategy carried out in this study, we were able to find nsSNPs with the potential for allelic change to affect coding of proteins and have a phenotypic effect.

## Conclusions

The *in silico* analysis identified nsSNPs that produce structural changes and decrease protein stability. Only the allelic change for rs209984404-HTR1B had a significant effect on the pen score evaluated in a Brahman population. The allele with loss of effective function of HTR1B confirmed a genotype of cattle with docility.

## Electronic supplementary material

Below is the link to the electronic supplementary material.

Supplementary Material 1

## Data Availability

Data is available on request to corresponding author.
