# Supplementary material for: Analysis of nonsynonymous SNPs in candidate genes that influence bovine temperament and evaluation of their effect in Brahman cattle
Source: Mol Biol Rep. 2024 Feb 7;51(1):285. doi: 10.1007/s11033-024-09264-4 (PMC10850011; doi:10.1007/s11033-024-09264-4)
Supplement: Supplementary file 1 — Supplementary Material 1 [file 11033_2024_9264_MOESM1_ESM.docx]

**Analysis of nonsynonymous SNPs in candidate genes that influence bovine temperament and evaluation of their effect in Brahman cattle**

Gilberto Ruiz-De-La-Cruz, Ana María Sifuentes-Rincón, Francisco Alejandro Paredes-Sánchez, Gaspar Manuel Parra-Bracamonte, Eduardo Casas, David G. Riley, George A. Perry, Thomas H. Welsh Jr. and Ronald D. Randel

**Supplementary material**

**Table Supplementary 1.** Candidate genes with nsSNPs were selected to evaluate whether they had a structural change in the protein.

| Gene | SNP ID | MQ | RF | AA | Change | POS |
| --- | --- | --- | --- | --- | --- | --- |
| SLC18A2 | Reference | GMQE  0.46 | 92.51% |  |  |  |
|  | rs110365063 | GMQE  0.44 | 90.75% | A/T | Yes | 63 |
| HTR1B | Reference | GMQE  0.75 | 96.33% |  |  |  |
|  | rs209984404 | GMQE  0.75 | 96.5% | A/S | Yes | 83 |
|  | rs722705037 | GMQE  0.66 | 96.44% | A/T | No | 60 |

Gene: gene abbreviation; SNP ID: Ensembl database identifier; MQ: modeling quality; RF: percentages of amino acids in favorable positions in the Ramachandran test; AA: exchange amino acids; POS: residue position of protein.
